# Supplementary material for: Red Mud Potentially Alleviates Ammonia Nitrogen Inhibition in Swine Manure Anaerobic Digestion by Enhancing Phage-Mediated Ammonia Assimilation
Source: Microorganisms. 2025 Mar 19;13(3):690. doi: 10.3390/microorganisms13030690 (PMC11944383; doi:10.3390/microorganisms13030690)
Supplement: Supplementary file 1 [file microorganisms-13-00690-s001.zip › Supplementary materials.docx]

**Supplementary materials for**

**Red mud potentially alleviates ammonia nitrogen inhibition in swine manure anaerobic digestion by enhancing phage-mediated ammonia assimilation**

Yulong Peng ^a, b^, Luhua Jiang  ^a, b^, *, Junzhao Wu ^a, b^, Jiejie Yang ^a, b^, Ziwen Guo ^a, b^, Manjun Miao ^a, b^, Zhiyuan Peng ^a, b^, Meng Chang ^a^, Bo Miao ^a, b^, Hongwei Liu ^a, b^, Yili Liang ^a, b^, Huaqun Yin ^a, b^, Qiang He ^c^, Xueduan Liu ^a, b^.

^a^ School of Minerals Processing and Bioengineering, Central South University, Changsha 410083, China

^b^ Key Laboratory of Biometallurgy of Ministry of Education, Central South University, Changsha 410083, China

^c^ Depa Supplementary figures lementary figures ntal Engineering, University of Tennessee, Knoxville, Knoxville, TN, USA.

* Corresponding authors. E-mail: [jiangluhua@csu.edu.cn](mailto:jiangluhua@csu.edu.cn)

The following are included as supplementary material for this paper:

Number of pages: 15

Number of texts: 2

Number of tables: 9

Number of figures: 2

### Texts S1: Exploring the optimal amount of red mud addition through the pre-experiment.

### The pre-experiment was performed in 250 mL anaerobic serum bottles, filled with 10 mL inoculum and 90 mL swine manure. Contents of 0.25%, 0.5%, 1%, 2%, and 4% of red mud were added, respectively, and the treatment groups without red mud were labeled as CK. After that, all other conditions and operations were the same as the batch experiments. The results of the pre-experiment are shown in Figure S1 and Figure S2.

Texts S2: Metagenomic analysis.

Total DNA was extracted from the precipitate using DNA extraction kits by Guangdong Magigene Biotechnology Co., Ltd. (Guangzhou, China). The integrity and purity of the DNA were assessed via 1% agarose gel electrophoresis. The extracted DNA were sequenced on the Illumina Novaseq Novaseq 6000 platform. After quality screening by Fastp, Initial sample, CK and RM obtained about 70 million, 77 million and 75 million clean reads, respectively, for de novo assembly by MEGAHIT. Prodigal software was used for ORF prediction.

| Mineral composition | Chemical formula | Percent |
| --- | --- | --- |
| Hematite | Fe_2_O_3_ | 43.33 |
| Sodalite | Na_4_(Al_3_Si_3_O_12_)Cl | 13.34 |
| Goethite | FeO(OH) | 12.53 |
| Quartz | SiO_2_ | 9.43 |
| Muscovite | KAl_2_(AlSi_3_O_10_)(OH)_2_ | 5.61 |
| Calcite | CaCO_3_ | 5.21 |
| Boehmite | AlOOH | 4.80 |
| Rutile | TiO_2_ | 4.04 |
| Gibbsite | Al(OH)_3_ | 1.71 |

Tables S1. Mineral composition of red mud

Tables S2. Primary characteristics of swine manure and inoculum.

| Parameter | Inoculum | Swine manure |
| --- | --- | --- |
| TS (g/L) | 23.7 | 24.7 |
| VS (g/L) | 5.8 | 7.7 |
| SCOD (mg/L) | 7800 | 11500 |
| concentration of total nitrogen (mg/L) | 266 | 327 |
| concentration of ammonia nitrogen (mg/L) | 211 | 278 |
| concentration of nitrate (mg/L) | 0.29 | 0.48 |
| pH | 6.48 | 6.56 |

Tables S3. General characteristics of bacterial metagenomic sequencing and assembly

| Sample | Total number | Total length (bp) | Average length  (bp) | Max length  (bp) | N50 length  (bp) | N90 length (bp) | GC(%) |
| --- | --- | --- | --- | --- | --- | --- | --- |
| RM.1 | 178.42 | 215256 | 155274 | 300 | 895 | 43.23 | 95.34 |
| RM.2 | 213.22 | 257025 | 244947 | 300 | 905 | 39.67 | 95.27 |
| RM.3 | 260.76 | 296263 | 202269 | 300 | 983 | 39.94 | 93.1 |
| CK.1 | 471.95 | 533455 | 222614 | 300 | 949 | 50.68 | 84.9 |
| CK.2 | 383.1 | 437420 | 161125 | 300 | 940 | 50.81 | 88.79 |
| CK.3 | 706.78 | 814468 | 221377 | 300 | 929 | 49.75 | 84.71 |

Tables S4. General characteristics of viral metagenomic sequencing and assembly

| Sample | Total base (Mb) | Number of contigs | Max  length | Min  length | N50 | GC (%) | Read used (%) |
| --- | --- | --- | --- | --- | --- | --- | --- |
| CK.1 | 178.42 | 215256 | 155274 | 300 | 895 | 43.23 | 95.34 |
| CK.2 | 213.22 | 257025 | 244947 | 300 | 905 | 39.67 | 95.27 |
| CK.3 | 260.76 | 296263 | 202269 | 300 | 983 | 39.94 | 93.1 |
| RM.1 | 471.95 | 533455 | 222614 | 300 | 949 | 50.68 | 84.9 |
| RM.2 | 383.1 | 437420 | 161125 | 300 | 940 | 50.81 | 88.79 |
| RM.3 | 706.78 | 814468 | 221377 | 300 | 929 | 49.75 | 84.71 |

Tables S5. Statistical analysis of the types of virus genomes identified

| Sample | Total | dsDNA (%) | ssDNA (%) | dsRNA (%) | ssRNA (%) | RT (%) | Unassigned(%) |
| --- | --- | --- | --- | --- | --- | --- | --- |
| MergedSample | 172656 | 132747  (76.89%) | 10481  (6.07%) | 90 (0.05%) | 472 (0.27%) | 1 (0%) | 28865 (16.72%) |

Tables S6. The alpha-diversity indexes of microbial communities at genus level

| Treatment | Shannon | Simpson | Chao1 |
| --- | --- | --- | --- |
| RM | 5.56 ± 0.11 | 0.179 ± 0.01 | 4142.67 ± 19.66 |
| CK | 5.60 ± 0.14 | 0.197 ± 0.02 | 4168.33 ± 18.32 |

The data in the table is mean ± SD

Tables S7. The alpha-diversity indexes of viral communities at contigs level

| Treatment | Shannon | Simpson | Chao1 |
| --- | --- | --- | --- |
| CK | 9.63 ± 0.96 | 0.986 ± 0.01 | 110401.67 ± 12874.42 |
| RM | 12.01 ± 1.46 | 0.995 ± 0.01 | 72171.25 ± 9568.95 |

The data in the table is mean ± SD

Tables S8. The RPKM values of nitrogen metabolism-related AMGs in CK and RM.

| KO ID | AMG | Sample | |
| --- | --- | --- | --- |
|  |  | CK | RM |
| K01915 | glnA, EC:6.3.1.2 | 60 | 108 |
| K00266 | gltD,EC:1.4.1.13 | 2 | 6 |
| K00262 | gdhA, EC:1.4.1.4 | 6 | 8 |
| K00260 | gudB, EC:1.4.1.2 | 1 | 1 |
| K00926 | arcC, EC:2.7.2.2 | 3 | 3 |
| K01673 | cynT, EC:4.2.1.1 | 1 | 1 |
| K00459 | ncd2, EC:1.13.12.16 | 8 | 13 |

Tables S9. Topological properties of the Phages-Host network analysis

| Treatment | Nodes | Edges | Average degree | Network density | Average path distance | Modularity | Eigenvector centrality |
| --- | --- | --- | --- | --- | --- | --- | --- |
| RM | 2962 | 39500 | 26.617 | 0.009 | 4.273 | 0.822 | 0.373 |
| CK | 3499 | 43373 | 24.792 | 0.007 | 4.323 | 0.8 | 0.361 |


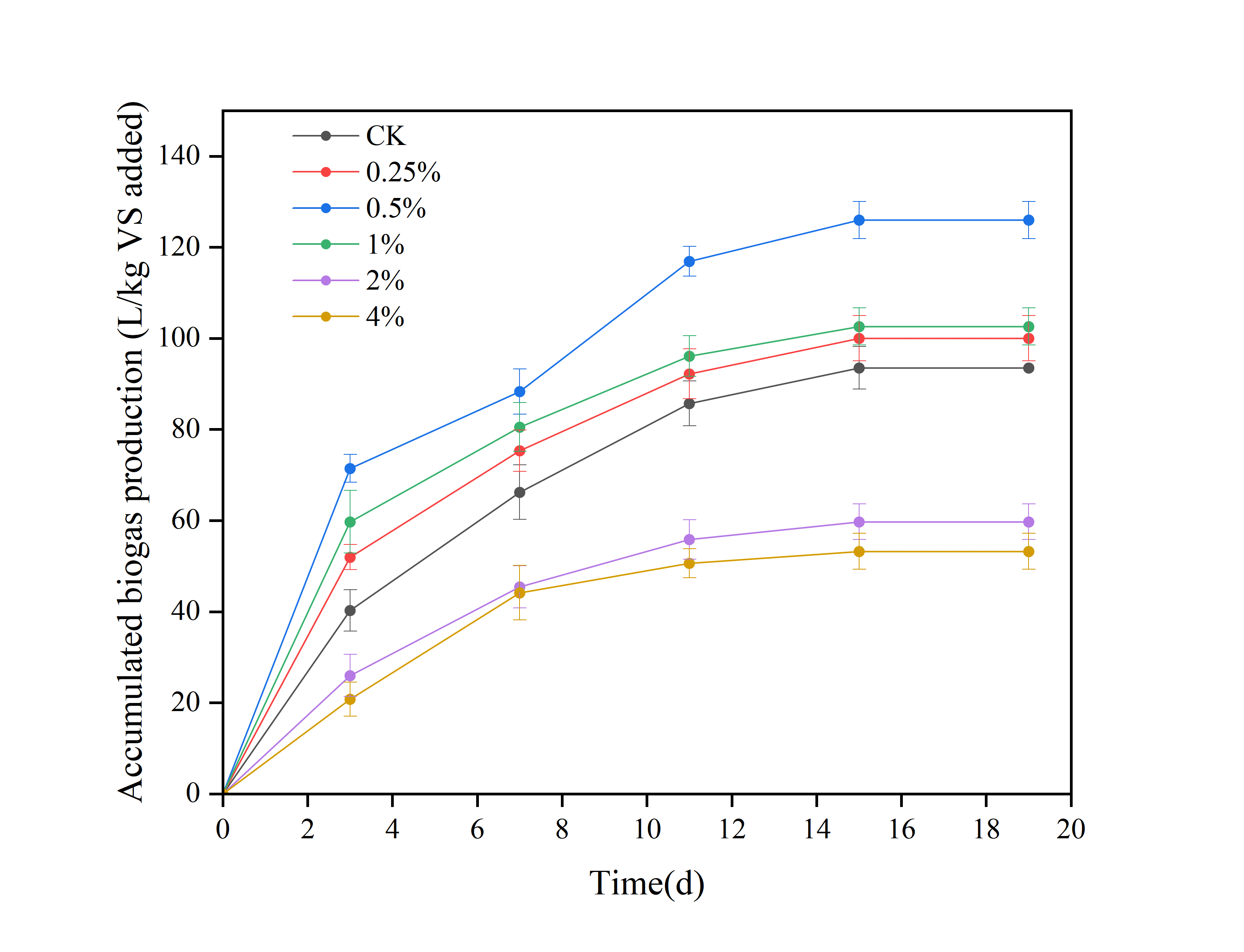


Fig. S1. Accumulated biogas production in the pre-experiment.


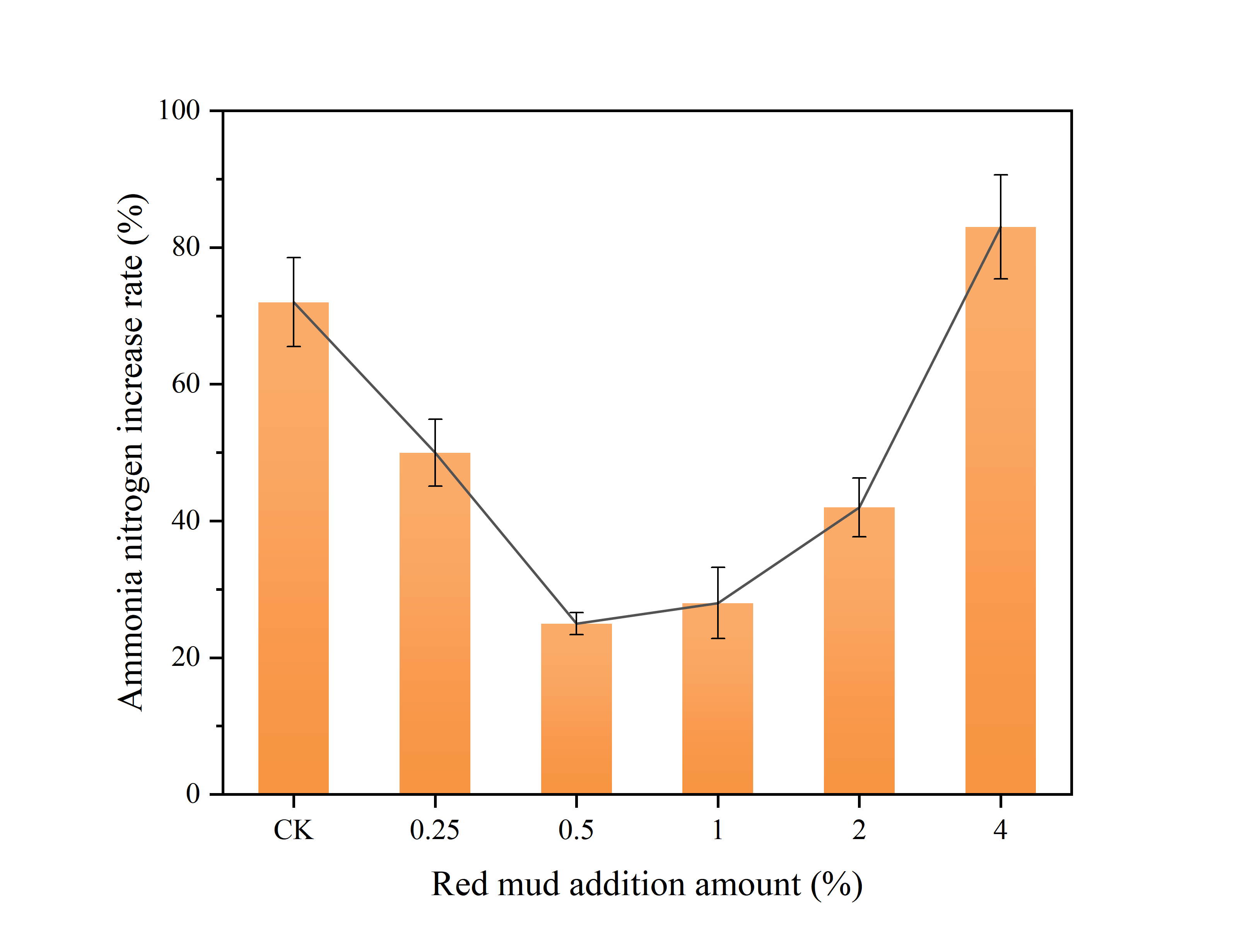


Fig. S2. Growth rate of ammonia nitrogen after anaerobic digestion in the pre-experiment
